# Intraguild predation in pioneer predator communities of alpine glacier forelands

**DOI:** 10.1111/mec.12649

**Published:** 2014-02-03

**Authors:** Lorna Raso, Daniela Sint, Rebecca Mayer, Simon Plangg, Thomas Recheis, Silvia Brunner, Rüdiger Kaufmann, Michael Traugott

**Affiliations:** * Institute of Ecology, University of InnsbruckTechnikerstraße 25, 6020, Innsbruck, Austria; † Meteoexploration.comHöttingergasse 21/17, 6020, Innsbruck, Austria

**Keywords:** Carabidae, Collembola, community assembly, Linyphiidae, Lycosidae, multiplex PCR, primary succession, trophic interactions

## Abstract

Pioneer communities establishing themselves in the barren terrain in front of glacier forelands consist principally of predator species such as carabid beetles and lycosid spiders. The fact that so many different predators can co-inhabit an area with no apparent primary production was initially explained by allochthonous material deposited in these forelands. However, whether these populations can be sustained on allochthonous material alone is questionable and recent studies point towards this assumption to be flawed. Intraguild predation (IGP) might play an important role in these pioneer predator assemblages, especially in the very early successional stages where other prey is scarce. Here, we investigated IGP between the main predator species and their consumption of Collembola, an important autochthonous alternative prey, within a glacier foreland in the Ötztal (Austrian Alps). Multiplex PCR and stable isotope analysis were used to characterize the trophic niches in an early and late pioneer stage over 2 years. Results showed that intraguild prey was consumed by all invertebrate predators, particularly the larger carabid species. Contrary to our initial hypothesis, the DNA detection frequency of IGP prey was not significantly higher in early than in late pioneer stage, which was corroborated by the stable isotope analysis. Collembola were the most frequently detected prey in all of the predators, and the overall prey DNA detection patterns were consistent between years. Our findings show that IGP appears as a constant in these pioneer predator communities and that it remains unaffected by successional changes.

## Introduction

Glacier forelands are the areas exposed as glaciers retreat. Most of the world glaciers have been steadily retreating for the past 150–200 years (Zemp *et al.* 2011), exposing more and more areas in front of them. The possibility of having a constant formation of freshly exposed sites, the fact that these areas are clearly delimited by the glacier's path and that they are in chronological sequence, make them ideal for succession studies. Early studies on invertebrates of glacier forelands have focused on the colonization and succession of species from late to early stages of deglaciation (Kaufmann 2001; Kaufmann *et al.* 2002; Hodkinson *et al.* 2004). One of the most interesting points that arises when looking at the earliest pioneer stages is the fact that at these stages of colonization, when there are practically no plants or apparent food sources, there is a high number of predatory invertebrates (Kaufmann 2001; Hodkinson *et al.* 2004). How these pioneer communities are able to exist in such a nutrient poor environment could not be answered so far.

One apparent food source is aeolian input, mainly from flying insects (reviewed in Hodkinson *et al.* 2002). Hodkinson *et al*. (2001) argue that, particularly in the Arctic islands of Spitsbergen, this could be one of the largest sources of nutrient input, as linyphiid spiders are able to trap these flying insects in webs that cover large areas of the forelands. However, Hawes (2008) found aeolian fallout to be composed primarily of organic detritus and points out that major aeolian fallout of arthropods may be rare events. Furthermore, not all glacier foreland systems appear to have high densities of linyphiid spiders as the ones in Spitsbergen which can capture this aeolian nutrient input. For example, alpine glacier forelands tend to be dominated by carabid beetles and lycosid spiders as the main predators in the earliest stages (Kaufmann 2001; Gobbi *et al.* 2006, 2007).

König *et al*. (2011) studied the food web of the glacier foreland arthropod community in the Rotmoos valley in the Austrian Alps using stable isotope analysis. They concluded that 2–34 years after deglaciation, predator diet was composed principally of decomposers (collembolans) whereas an increase in cannibalism or intraguild predation was suggested by their data for later stages. Collembola, an important food source for invertebrate predators (Bilde *et al.* 2000; Agustí *et al*. 2003; Eitzinger & Traugott 2011), are already present close to glacier snouts, and they appear throughout the glacier foreland (Kaufmann *et al.* 2002; König *et al*. 2011). However, as König *et al*. (2011) suggest, these detritivores are not necessarily the only food sources of the main predators, which might also consume other predatory species. This so-called intraguild predation (IGP) combines competition and predation, as species from the same feeding guild feed on each other (Polis *et al.* 1989). Invertebrate generalist predators typically display IGP, and it has been studied extensively in relation to biological control as it can diminish pest regulation under certain circumstances (Rosenheim *et al.* 1995; Hodge 1999; Janssen *et al.* 2006; Traugott *et al.* 2012; Davey *et al.* 2013). Polis & McCormick (1987) found that in scorpion populations, IGP increased when food availability was low. This gives survivors a double advantage, as are they not only feeding but also reducing the number of competitors. It can therefore be expected that IGP is high in the earliest predator assemblages of the glacier forelands, when the community is composed of opportunistic predators, vegetation is absent, and other food sources are scarce. In the later pioneer stages, with an initial vegetation already present, the increase in alternative prey should lead to a reduction in IGP. In this study, we tested this hypothesis in two consecutive years by assessing the levels of IGP within a community of pioneer predators occurring in the glacier foreland of the Rotmoosferner, situated in the Ötztal valley in Austria. The macroinvertebrate predator community at the glacier edge comprises carabid beetles, wolf and linyphiid spiders as well as one species of harvestman (Kaufmann 2001).

Molecular techniques have become one of the methods of choice when looking at feeding interactions among invertebrates (Symondson 2002; Greenstone & Shufran 2003; Pompanon *et al.* 2012; Traugott *et al.* 2013). Problems associated with classical gut content analysis such as the difficulty to identify partially digested or soft-bodied prey, predators that are fluid feeders or small, can be overcome with molecular methods (Sheppard & Harwood 2005). Multiplex PCR enables to screen large numbers of dietary samples simultaneously for several prey taxa, using species- and/or group-specific primers (Harper *et al.* 2005; Sint *et al.* 2012). Marrying molecular methods to stable isotope analysis makes it possible to get the best of both worlds: detailed information from the DNA-approach together with an assessment of the assimilation of broader categories of resources and the trophic level of the consumers which is reflected in the animals' carbon and nitrogen signatures, respectively (Carreon-Martinez & Heath 2010; Hardy *et al.* 2010; Traugott *et al.* 2013).

During 2009 and 2010, we analysed predator isotopic signatures as well as the consumption of intraguild and collembolan prey in the arthropod predators. Early (≤7 years ice-free) and late (12–20 years ice-free) pioneer stages were compared in the Rotmoos glacier foreland to address three questions:

1. Is the detection frequency for IGP higher in the early than in the late pioneer stage?

It would be plausible to hypothesize that as between early and late pioneer stages there are differences in the available food resources and in the arthropod community composition, entailing effects on the magnitude of IGP. For example, in the late pioneer stage, a higher abundance and higher diversity of alternative prey are expected due to an increased plant cover and consequently increased availability of detritus, thereby reducing IGP between predators.

1. Compared to the predation on Collembola, is IGP a frequent feeding interaction for these predators and is the interaction symmetrical or asymmetrical?

In this study, we compare the frequency of IGP to predation rates on Collembola, the latter being an example of a probably important prey group for the pioneer predators (see above). Furthermore, IGP can be either symmetrical (all predators feed on each other) or asymmetrical in case certain intraguild predators consume other predators (so-called intraguild prey) and when the latter do not feed on the former (Polis *et al.* 1989).

1. Do the predator species utilize similar intraguild prey or do they occupy their own trophic niche?

The carabid and arachnid predator species in the glacier foreland are likely to have a different prey choice due to a taxon-specific feeding behaviour. However, due to a comparably small pool of available prey, the predator taxa might be forced to utilize the resources in a similar way and consequently occupy a similar feeding niche. Here, we look at this aspect by comparing niche overlap in IGP and collembolan prey among carabid beetles and arachnids in both early and late pioneer stages.

## Materials and methods

### Study site

The Rotmoos glacier valley is northwest facing in the upper Ötztal valley near Obergurgl, Tyrol, Austria (46.826 N, 11.046 E). Two areas were chosen for study: the area representing the early pioneer stage was closest to the glacier (0–70 m), has been ice-free for the last 0–7 years, is at an average altitude of 2436 m a.s.l. and covers an area of approximately 7500 m^2^. Vegetation was scarce (overall cover 2%), and the area is irregular terrain, covered with inorganic material ranging from fine sand to large boulders. The area representing the late pioneer stage was 100–150 m away from the glacier, has been ice-free for the last 12–20 years, is at an average altitude of 2418 m a.s.l. and covers an area of approximately 6000 m^2^. Vegetation cover was on average 15% and is thus greater than in the early pioneer stage, and it also had a more stabilized substrate with a higher diversity of habitats such as fine-grained deposits from the glacier outflow. Both pioneer stages represent the earliest stage of plant succession according to Nagl & Erschbamer (2010), typical pioneer species are *Linaria alpina*, *Saxifraga oppositifolia* and *S. aizoides*.

### Fieldwork

The two pioneer sites in the Rotmoostal foreland were sampled for two consecutive years during the summer at the beginning of the snow-free period from the 4th of July to the 13th of August 2009 and from the 7th of July to the 10th of August 2010. Two grids of pitfall traps were set up with 10 m spacing between traps in the compass directions, one with 75 traps in the early pioneer stage and another one with 53 traps in the late pioneer stage. The reason for the disparity in number was we tried to ensure that the traps covered the valley floor between the side moraines the Rotmoos glacier left behind when retreating, resulting in different areas being covered. The pitfall traps were made by placing two plastic cups (diameter 9 cm), one inside the other, in a hole dug in the ground. The outer cup was used to maintain the trap hole in the ground when the inner cup was removed to empty the caught arthropods. The rim of the cups was at ground level and each trap had some wood chip added to it so as to provide cover for the trapped individuals. A flat stone, taken from the surroundings, was placed above the trap, leaving a space of approximately 5 cm so that arthropods were able to crawl in while ensuring the trap was protected from rain. Each area was sampled on alternative days. On the days in which there was no sampling, the traps were inactivated by placing a small piece of netting, which hung down the side of the trap, providing an escape route for accidental catches. Traps were left open for 24 ± 1 h and all trapped invertebrates were collected alive. Linyphiid spiders were not common in the traps, so extra samples were collected manually. In 2009, six wet pitfall traps (diameter 7 cm) per pioneer stage were installed adjacent to the trapping grid to record the activity of epigaeic arthropods. Traps were filled with 50% ethylene glycol and emptied fortnightly at 31 July and 14 August 2009. The caught arthropods were transferred to ethanol and identified to the lowest taxonomic level possible.

All the arachnids and carabid beetles collected in the field were placed individually in reaction tubes inside cooling bags and transported to the laboratory in the Alpine Research Centre in Obergurgl. Catches were dominated by four carabids (*Nebria germari*, *Nebria jockischii*, *Nebria rufescens* and *Oreonebria castanea*), two lycosids (*Pardosa nigra and Pardosa saturatior*), several linyphiids and one species of harvestman (*Mitopus glacialis*). All of these arthropods were used for dietary analysis and identified alive, except for the two species of wolf spiders which were assigned as *Pardosa* spp. and the linyphiid spiders which were identified at family level only. On arrival, all beetles were transferred to 150-mL beakers with damp tissue paper and placed in a 4 °C fridge. The reaction tubes in which carabid beetles had been transported were checked for regurgitates or faeces (a common occurrence). If a regurgitate was present, 435 μL of TES (0.1 mol TRIS, pH 8, 10 mmol EDTA, 2% sodium dodecyl sulphate) buffer and 5 μL of proteinase K (20% conc.) were added and the samples were stored at −24 °C. All spiders and harvestmen were numbered separately, and even numbers were frozen at −24 °C while the remaining odd numbered arachnids were stored in the beakers with damp tissue paper at 4 °C. In 2010, five lycosid spiders and eight individuals of each of the four species of carabid beetles (exception *N. rufescens* 10 individuals for late pioneer stage) where frozen from each pioneer stadium for stable isotope analysis. All live animals were released the next day in the areas where they had been taken from.

### DNA extraction and molecular analysis

DNA was extracted from all samples using a CTAB-based technique as described by Juen & Traugott (2005), although alterations were made depending on the nature of the samples. Spiders were homogenized in the same type of lysis buffer as the beetle regurgitates (see above) using 2-mm glass balls and a Precellys 24 tissue homogenizer (PEQLAB, Erlangen, Germany). All samples were incubated at 56 °C, beetle regurgitates for 3 h and spiders overnight, followed by the CTAB extraction protocol. Two extraction negative controls were included in each batch of 30 samples to check for carry-over contamination. The DNA extracts of the lycosid spiders contained inhibiting substances after the CTAB extraction; therefore, they were additionally subjected to the QIAquick PCR Purification Kit (Qiagen). All spider and regurgitate extracts were tested for prey DNA using a multiplex PCR system described in detail in Sint *et al*. (2012). This assay targeted intraguild (the four carabid species, *M. glacialis* and *Pardosa* spp.) and extraguild prey via a group-specific primer for Collembola. High concentrations of predator DNA, which are usually present in whole-body DNA extracts, can decrease the detection sensitivity of prey DNA when the multiplex PCR contains a primer pair targeting the predator (Traugott & Symondson 2008). In the current study, this situation applies to the lycosid spider samples; therefore, the concentration of the primer pair for *Pardosa* spp. was reduced from 0.2 to 0.04 μm for testing the lycosid samples. As the beetle regurgitates contained significantly less consumer DNA compared to full body extracts of the wolf spiders, no negative effect on prey detection was expected and the primer concentration for the respective predator was left unaltered.

### Stable isotope analysis

Carabid beetles and wolf spiders were also examined using stable isotope analysis. Wing covers from *N. germari*, *N. jockischii*, *O. castanea* and *N. rufescens* were dissected and used for stable isotope analysis. Gratton & Forbes (2006) found that different body parts can give different signatures. Wing covers were chosen after preliminary tests were made using wing covers, wings (*N. jockischii* and *N. rufescens*) and legs, and finding that similar results were obtained with all three body parts. *Pardosa* spp. had the outer segments of the legs removed (patella, tibia, tarsus and metatarsus) so as to ensure there was no contamination from the gut extending into the coxa and femur. All body parts were dissected with great care to ensure no cross-contamination between samples. Samples were then dried at 60 °C for a minimum of 48 h and weighed into tin capsules to the nearest 0.001 mg. Isotopic content in ^13^C and ^15^N was analysed in each sample by the Kompetenzzentrum Stabile Isotope, Forschungszentrum Waldökosysteme, University of Göttingen (Germany). Pee Dee Belemnite marine limestone (PDB) for ^13^C and atmospheric nitrogen for ^15^N served as the primary standard; every four samples one acetanilide (C_8_H_9_NO) sample was added for internal calibration. The mean standard deviation of samples of 10–200 mg N, representing the range of nitrogen in our samples, was 0.15‰ (Werner *et al.* 1999). Isotopic abundance is expressed by the isotopic ratio, δX (‰) = (*R*_sample_ − *R*_standard_)/*R*_standard_ × 1000, where X represents ^13^C and ^15^N, and *R*_sample_ and *R*_standard_ represent the ^13^C/^12^C and ^15^N/^14^N ratios of the sample and standard, respectively.

### Statistical analysis

Differences in prey DNA detection rates were compared between the predators in the early and late pioneer stages and between years 2009/2010 by calculating the 95% confidence interval using the exact binomial test (Clopper & Pearson 1934). The ‘exact’ method of Clopper and Pearson has often been regarded as definitive; it eliminates both aberrations and guarantees strict conservatism (Newcombe 1998). DNA detection rates were pooled for each predator taxon and were compared between years and areas. Nonoverlapping confidence intervals were interpreted as significant differences at *P* < 0.05. Fisher's exact test, which has no restrictions for small data samples (e.g. Agresti 2002), was used to test for significant differences between specific feeding links in the early and late pioneer stages.

Niche overlap was calculated by using Pianka's index:
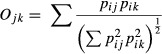
1 where *p*_*ij*_ and *p*_*ik*_ are the frequency of occurrence of prey item *i* in the diet of species *j* and *k,* respectively (Pianka 1973). Pianka's index gives a measure for the relative amount of niche overlap. The niche occupied by each species is compared for each species against each other. Values of 1.0 indicate that they occupy the same niche (share exactly the same resources), and values of 0.0 mean that there is no similarity in the niche occupied (they share no resources). When calculating Pianka's index, the data were pooled for 2009 and 2010 and the prey DNA detection frequencies were used for the calculation. Note that the detection frequencies used here do not sum up to 100%, and therefore, this is not exactly Pianka's index for diet composition which, however, does not impair its meaning as an overlap index.

To look at the species niche overlaps, a dendrogram from UPGMA-clustering was arranged according to ordination axis 1 from a principal coordinate analysis (PCO). The correlation of the ordination axis with the prey items is also represented. Mean values of ^13^C and ^15^N were compared between species with anova (post hoc contrasts Bonferroni corrected), and Levene's test was used to check the stable isotope data for homoscedasticity between species. Pairwise F-tests (Bonferroni corrected) were conducted post hoc to identify heteroscedastic groups. Graphs and statistical analyses were conducted using R (R Development Core Team 2012), mvsp 3 (used for cluster analysis, Kovach Computing Services 2010), Canoco 5 (used for PCO, Biometris & Šmilauer 2012) and ibm spss Statistics 20.

## Results

The average catch (±SEM) of ground-dwelling arthropods in early/late pioneer stage per wet pitfall trap and day was the following: *Nebria germari* 0.93 ± 0.12/0.31 ± 0.11, *N. jockischii* 0.26 ± 0.14/0.28 ± 0.22, *N. rufescens* 0.00 ± 0.00/0.012 ± 0.012, *Oreonebria castanea* 1.09 ± 0.19/1.37 ± 0.66, other Carabidae 0.07 ± 0.05/0.32 ± 0.14*,* other beetles 0.08 ± 0.03/0.48 ± 0.15*,* beetle larvae 0.04 ± 0.02/0.04 ± 0.03*, Pardosa* spp. 0.29 ± 0.08/0.27 ± 0.05, *Mitopus glacialis* 0.20 ± 0.04/0.20 ± 0.08, Linyphiidae 0.018 ± 0.012/0.071 ± 0.016, Diptera 0.41 ± 0.10/0.73 ± 0.13, Plecoptera 0.006 ± 0.006/0.226 ± 0.149, Collembola 9.83 ± 1.32/2.21 ± 0.80 and Acari 0.23 ± 0.05/1.18 ± 0.34.

**Figure 1:**
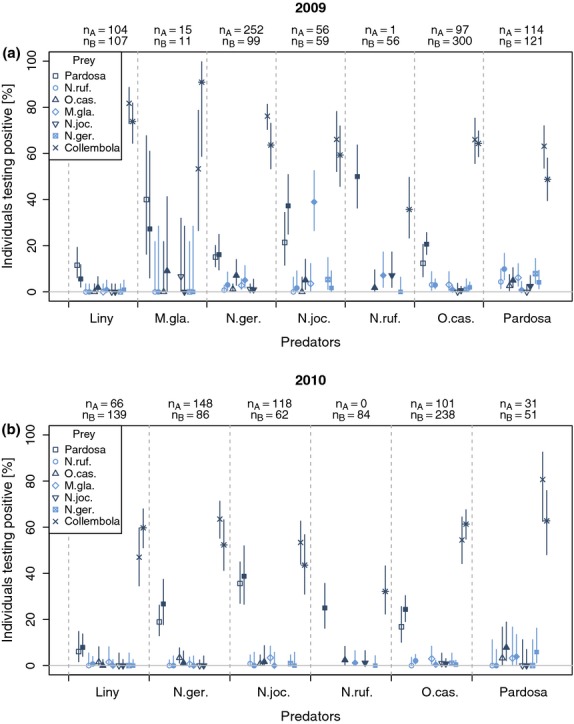
Percentage of the number of predators testing positive for each type of prey for the year 2009 (a) and 2010 (b). For each species, the results for the early pioneer stage are represented by the empty symbols and the late pioneer stage with the full symbols (+ = ⊕, x = *). Error bars represent the 95% interval calculated using a binomial exact test. At the top of each section, the total number of a particular taxon caught in each area is given. nA = Total number of individuals caught in the early pioneer stage, nB = Total of individuals caught in the late pioneer stage. Taxa names: Liny = Linyphiidae, M.gla = *Mitopus glacialis*, N.ger = *Nebria germari*, N.joc = *Nebria jockischii*, N.ruf = *Nebria rufescens*, O.cas = *Oreonebria castanea*, and Pardosa = *Pardosa* spp.

The prey DNA detection rates showed a consistent pattern between years with all predators having Collembola as the most frequently detected component of their diet with detection rates ranging between 35% and 90% (Fig.1). Collembola were detected significantly more often than other prey in the diets of all the predators except for *N. jockischii* and *N. rufescens*. *Pardosa* spp. DNA was present in the diets of all the *Nebria* species, and especially in *N. rufescens* and *N. jockischii,* a high percentage of the tested specimens (25–60%) contained this type of prey, being the most common intraguild prey item found (with the exception of *N. jockischii* in the late pioneer stage in 2009; Fig.1). However, in the wolf spiders, the most frequently detected DNA was from Collembola with only a small percentage of the individuals testing positive for intraguild prey, that is, the four carabid species (Fig.1). *Nebria rufescens* and *N. jockischii* were the two species with the highest detection frequency of intraguild DNA in their regurgitates, particularly in 2009. In linyphiid spiders, Collembola DNA was detected more often in relation to intraguild prey than in any other predator species, that is, very few individuals contained wolf spider or carabid DNA. In the few *M. glacialis* samples, the most frequent prey group detected was Collembola, followed by *Pardosa* spp. and some *N. germari* and *N. jockischii* (Fig.1).

For the 2010 data, no significant differences in intraguild and extraguild feeding rates between the early (*E*) and late (*L*) pioneer stages were found in all predators examined in all cases (*P* > 0.05). However, in 2009, DNA of *M. glacialis* and of Collembola was detected significantly less frequently in the wolf spiders caught in the late compared to that of the early pioneer stage (*M. glacialis E* = 6.14%, *L* = 0.83%, *P* = 0.031; Collembola *E* = 63.16%, *L* = 48.76%, *P* = 0.035). In *N. germari* captured in the late pioneer stage, *O. castanea* was detected significantly more often (*E* = 1.19%, *L* = 7.07%, *P* = 0.007), but Collembola was detected significantly less often (*E* = 76.19%, *L* = 63.64%, *P* = 0.023) compared to those found in the early stage. In *N. jockischii*, DNA of *M. glacialis* was detected significantly more often in the late compared to the early pioneer stage (*E* = 3.57%, *L* = 38.98%, *P* < 0.001). All other feeding interactions were not significantly different between the two successional stages.

**Figure 2:**
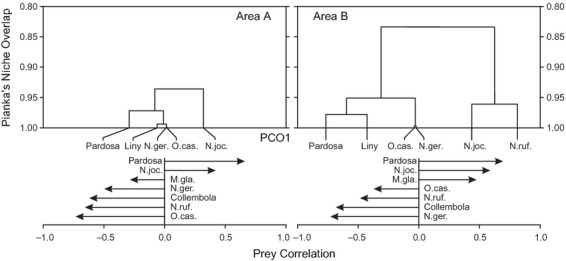
Dendrograms of Pianka's niche overlap for early and late pioneer stages on a common scale with the predators arranged according to the PCO's ordination axis 1. In addition, the correlations of the ordination axis with the prey items are included.

In both early and late pioneer stage, values for the niche overlap for the IGP and collembolan feeding events were all above or close to 0.8, indicating that all the predators were sharing similar intraguild prey resources (Fig.2). However, in the late pioneer stage, there was a reduction in the index for the *N. jockischii*–*Pardosa* relationship, signalling a possible shift towards more diverse feeding. It appeared that *N. rufescens* had the most differentiated diet in relation to the other species, although it was still similar to that of *N. jockischii* (Fig.2). *Nebria rufescens* was not included for the calculation in the early pioneer stage as only one individual was found in that stage. The dendrogram (Fig.2) shows that the smaller carabid species, *N. germari* and *O. castanea*, utilize similar resources. In the late pioneer stage, this also appeared to be the case for *N. jockischii*, *N. rufescens* and *Pardosa* spp. and the linyphiid spiders. The prey correlation also demonstrates the overall importance of Collembola and *Pardosa* spp. as prey in both pioneer stages (Fig.2).

**Figure 3:**
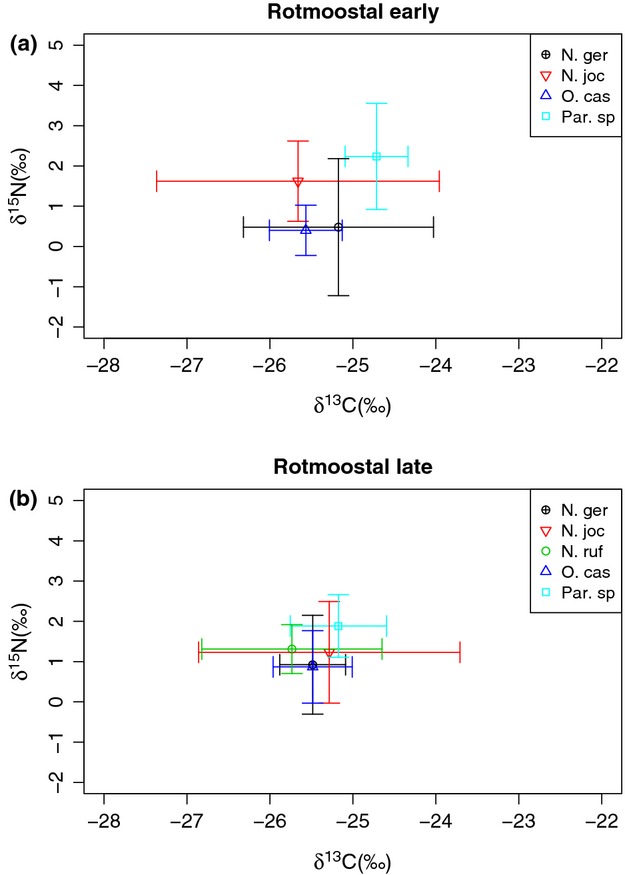
Mean delta ^15^N and ^13^C signatures (±1SD) for the carabids and wolf spiders collected in the Rotmoos glacier foreland in early (a) and late (b) pioneer stages. Species names: N. ger = *Nebria germari*, N. joc = *Nebria jockischii*, N. ruf = *Nebria rufescens*, O.cas = *Oreonebria castanea*, and Par. sp = *Pardosa* spp.

In general, the isotopic signatures in carbon and nitrogen showed high similarity between the carabids and arachnids in both pioneer stages (Fig.3). No significant differences were found within any of the species between the early and late pioneer stage; thus, the two successional stages were pooled for comparisons between species. The isotopic signature in nitrogen differed significantly between the five predatory species (*F*_4, 66_=3.87, *P* = 0.007), and the post hoc tests revealed that the δ^15^N signal of *Pardosa* spp. was significantly higher than the one of *N. germari* (*P* = 0.022) and *O. castanea* (*P* = 0.014). No significant differences were found between the species regarding their mean carbon isotopic signature.

Carbon isotopic signal is indicative of the food sources used, and Levene's test showed that significant differences in the variances of the predators' δ^13^C signal (*P* = 0.004) occurred. Pairwise comparisons via F-tests showed that the variance in δ^13^C was significantly larger in *N. jockischii* and *N. rufescens* compared to *O. castanea* (*P* < 0.01 and *P* = 0.02 after Bonferroni correction, respectively). The same was true for the comparison between *N. jockischii* and *Pardosa* spp., where the carbon signal was significantly wider in the former than the latter (*P* = 0.02 Bonferroni corrected). For the δ^15^N signal, no significant effect was found regarding the variance of the data.

## Discussion

Contrary to our initial hypothesis, we were not able to detect a clear difference in the prey detection frequencies of the predators between the early and late pioneer stages in the 2 years investigated. There were only significant differences in the feeding patterns between early and late pioneer stage in *Pardosa* spp., *Nebria jockischii* and *N. germari,* but none of them was in line with our hypothesis as the intraguild prey DNA detection rates were higher in the late compared to the early pioneer stage. We assumed that there would be more IGP in the earlier pioneer stage due to the lack of nonintraguild prey as confirmed with the wet pitfall traps in 2009. However, our work demonstrates that Collembola are an important prey resource right at the start of the glacier foreland succession, as predators caught in the traps closest to the glacier snout frequently tested positive for them. The overall high detection rates of collembolan prey in all of the pioneer predators in the current study supports the notion of König *et al*. (2011) ‘that the formation of terrestrial food webs during early primary succession heavily relies on prey out of the decomposer system’.

Our results also suggest that in the Rotmoos glacier foreland, IGP interactions are symmetrical according to the definition of Polis *et al*. (1989) as, apart from the linyphiid spiders, all predator species fed on each other to a certain extent. However, the carabid species were more frequently consuming other intraguild prey (especially wolf spiders) than were the spiders. *Nebria jockischii*, the largest of the four carabid species investigated here, seems to act as the top intraguild predator, being one of the most diverse feeders with a high proportion of lycosid prey but with very few other predators feeding on it.

The diurnal or nocturnal behaviour of the predators should be taken into account when explaining the observed intraguild interactions. *Nebria jockischii* and *N. rufescens* are known to be both diurnal and nocturnal (Kaufmann & Juen 2001; Gereben-Krenn *et al.* 2011), while *N. germari* and *Oreonebria castanea* are considered to be nocturnal, although *N. germari* has also been spotted during the day (Gereben 1995). *Pardosa* spp. are diurnal as are the harvestmen (personal observation). The more likely encounter between *N. jockischii*/*N. rufescens* and *Pardosa* spp. during the day could also be a reason for the higher rate of predation on the wolf spiders. It is also important to consider that IGP does not necessarily have to be on adults because beetle larvae, spiderlings and eggs will also be detected in the guts and all these predators reproduce in the glacier foreland (Kaufmann & Juen 2001).

Overall, the current results from the stable isotope analysis are consistent with those of König *et al*. (2011), with all carabid and arachnid species clearly falling into the predator guild. Although stable isotope analysis integrates the diet over larger time spans, only food sources which differ in their isotopic composition can be differentiated, providing it with a low resolution on what has been consumed specifically (Boecklen *et al.* 2011). Furthermore, the absence of isotopic variability in ^13^C among the prey available to the pioneer predators, as indicated by the study of König *et al*. (2011), might explain their poor isotopic separation. However, the variability of the ^13^C signal of *N. jockischii* and *N. rufescens* was larger than in the other predators. As summarized by Bearhop *et al*. (2004), ^13^C can suggest food sources from different origins just as ^15^N variation is a good proxy for changes in trophic position. In this case, we could hypothesize that *N. jockischii* and *N. rufescens*, the two largest carabids in this pioneer community, are feeding on a wider range of prey which may also originate from different habitats. Those two species are known to preferably occur along glacier streams (Kaufmann & Juen 2001) and thereby might also consume aquatic prey such as chironomids, simulids and stoneflies which have a significantly depleted ^13^C signature compared to terrestrial invertebrates found in this glacier foreland (König *et al*. 2011). The significantly lower variability in ^13^C found in *Pardosa* spp., and the two smaller carabid species *O. castanea* and *N. germari* points towards a narrower prey range. This interpretation is supported by general theory on predator–prey body mass relationships, suggesting that small predators have narrow diets while large predators feed on a wide range of prey and occupy higher trophic levels (Woodward & Hildrew 2002). On the other hand, carabid and harvestmen are known to readily feed on dead prey (Morse 2001; Foltan *et al.* 2005) and spiders can also be scavengers, particularly if the prey has not been dead for too long (Cramer 2008; Von Berg *et al.* 2012). This means that smaller predators are able to feed on larger prey, relaxing the size relationships in predator–prey interactions to some extent.

Niche differentiation analysis, based on molecular prey detection, also suggests that small carabids, spiders and large carabids differ in their trophic niche. The reduction in niche overlap which was observed in the late pioneer stage could be the result of an increase in species richness (Winemiller *et al.* 2001), suggesting that with an increase in prey choice, the predators are able to start to specialize and be more selective. Whether this development continues over a longer time span and is then even more pronounced on older areas with higher species richness would be an interesting topic to examine in further investigations. Furthermore, it is important to note that these results are based only on the IGP and collembolan data and it would be necessary to include the whole prey spectrum of the pioneer predators to reach more definitive conclusions.

There are a number of things that need to be considered when interpreting the present findings such as secondary predation (Sheppard *et al.* 2005). In our system, the main source of secondary prey most likely is collembolans. However, for all tested predators, Collembola DNA was rarely detected together with that of another prey (∼10% of samples). It is therefore unlikely that secondarily predated prey affected our results considerably. Cannibalism also goes undetected by our molecular prey detection system, which does not differentiate between DNA of the same species. However, spiders are known to be cannibalistic (Wagner & Wise 1996; Vanacker *et al.* 2004; Rypstra & Samu 2005); therefore, the significance of intraguild prey might have been underestimated by the current molecular data. This suspicion is supported by the comparably high ^15^N signature of *Pardosa* spp., indicating a significant share of intraguild prey in their diet. However, according to the molecular data, the lycosids were rarely feeding on carabid beetles or the harvestman, which suggests that their high ^15^N signal was derived by feeding on congeners. It is also important to keep in mind that gut content prey DNA detection rates do not necessarily equate to the proportion of each prey consumed. For example, although the high detection rates for Collembola, the only extraguild prey examined in the current study, show that this prey is frequently consumed, we cannot conclude with certainty that collembolans are the most significant prey in the overall diet.

Predator mobility between areas does not seem to be a source of error as previous mark–recapture experiments showed 1.5–6 m mean daily movement and hardly any recaptures beyond distances of 50 m (Baldes 2009; Brunner 2011). Predation events occurring within the traps when potential prey is exposed to predators will bias the trophic analyses as these feeding interactions might not happen under natural conditions (King *et al.* 2008). This source of error is also unlikely to have affected the current data as we used wood chips in all pitfall traps as shelter and to allow caught animals to hide from each other. Moreover, prey remains potentially stemming from such intratrap predation events were rarely observed, that is, during the overall 2009 sampling campaign only in 19 occasions such remains were found.

We can conclude that intraguild prey plays an important part in the diet of the predators colonizing the pioneer stages in the glacier foreland, but autochthonous detrital prey in the form of Collembola is an important source of food too. Contrary to our initial belief, the detection frequency of intraguild prey was not lower in the late compared to the early pioneer stage, suggesting that in these early successional stages, the levels of IGP stay constant. This view is supported by the ^15^N data which were not significantly altered between predators collected in early and late pioneer stage. However, the predators separated into different trophic niches with large carabid beetles acting as intraguild predators with a wider dietary range and small carabid beetles showing a more restricted diet while wolf spiders serve as intraguild prey for the larger carabids.

## Data accessibility

Data files and R script: Dryad doi:10.5061/dryad.6ph1c.
